# Post-partum cerebral venous sinus thrombosis: A case report

**DOI:** 10.5339/qmj.2024.13

**Published:** 2024-03-11

**Authors:** Shruti Jain, Mehak Bhushan, Vandana Talwar

**Affiliations:** 1 Department of Anaesthesiology and Intensive Care, Vardhman Mahavir Medical College & Safdarjung Hospital, New Delhi, India Email: shruti.anaesth@gmail.com

**Keywords:** Pregnancy, Puerperium, Thrombosis

## Abstract

Introduction: Cerebral venous sinus thrombosis (CVST) is a rare and life-threatening condition that may be encountered during pregnancy and puerperium. The diagnosis of CVST is a challenge because of its varied presentation.

Case Report: A 28-year-old woman presented with headache, projectile vomiting, and generalized tonic–clonic seizures 10 days after delivery by cesarean section. She had an uneventful antenatal period of 38 weeks of gestation. High clinical suspicion and the availability of magnetic resonance venography helped in making a diagnosis of CVST. She was successfully managed with a low-molecular-weight heparin (LMWH) and anti-epileptic therapy with no residual complications.

Discussion: Pregnancy induces several prothrombotic changes in the coagulation system that predispose to CVST. These changes persist for six to eight weeks after birth. Infection and cesarean section are the additional risk factors for CVST during puerperium. The symptoms of CVST depend on the sinuses and veins involved, raised intracranial pressure, and the extent of brain parenchymal injury.

Conclusion: Greater awareness of the disease and the availability of imaging modalities have contributed to the early diagnosis and favorable outcomes in these cases. LMWH is the main stay of treatment in this disease.

## Introduction

Cerebral venous sinus thrombosis (CVST) is a rare type of stroke that usually affects young individuals.^1^ Pregnancy and puerperium are the important risk factors for CVST.^2^ It is more common in women than in men in young and middle-aged populations.^3^

It is caused by complete or partial occlusion of the major cerebral venous sinuses or the smaller feeding cortical veins (cortical vein thrombosis). CVST presents with varied symptoms ranging from headaches to seizures and stroke.^3^ It is important to exclude conditions such as eclampsia and post-dural puncture headache (PDPH) in pregnant and postpartum women which may mimic the symptoms or may co-exist with CVST.^4,5^ Early diagnosis and treatment are important for a favorable outcome.

Here we report a case of CVST in a post-partum woman managed at a tertiary care center in a developing country after obtaining patient consent to publish this case report.

## Case Report

A 28-year-old post-partum woman, gravida1, para1, live1, presented to the emergency department of our hospital with a history of generalized tonic–clonic seizures.

**Figure 1.**
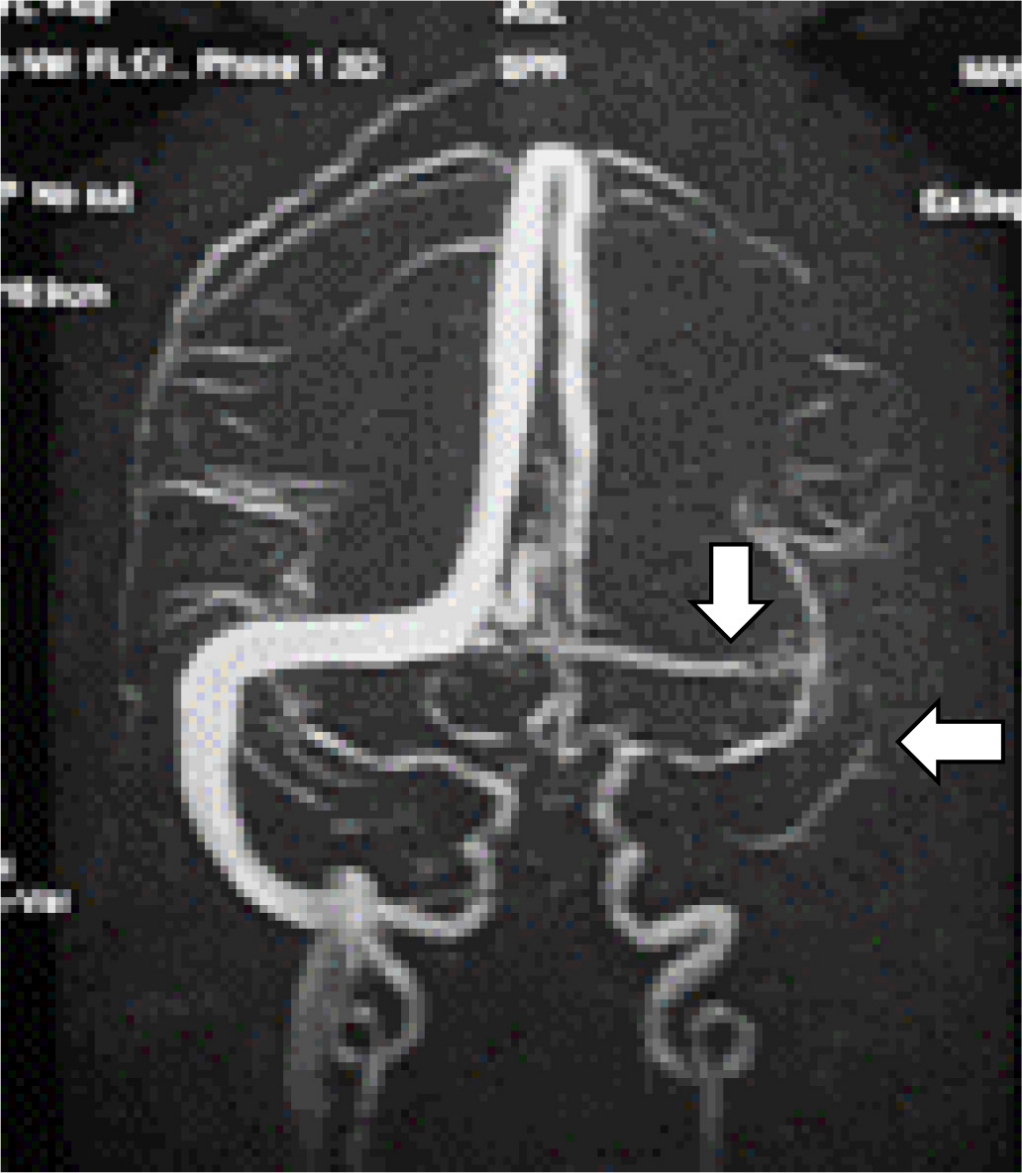
Magnetic resonance venography of the brain showing thrombosis of the sigmoid and transverse sinuses (arrows).

The patient had an uneventful antenatal period of 38 weeks of gestation, following which she had undergone a planned cesarean section under spinal anesthesia due to a constricted pelvis. Her antenatal records reported normal blood pressure throughout the pregnancy and the postpartum period. She was discharged on the third postoperative day. She was apparently well till the 10th postoperative day when she developed a sudden-onset generalized headache associated with nausea and projectile vomiting. The headache gradually increased in intensity and was not relieved by over-the-counter analgesics. It was not associated with fever, rash, ear discharge, blurred vision, neurological deficit, loss of consciousness, or seizures. After two days of persistent headaches, she had five episodes of generalized tonic–clonic-type seizures at intervals of approximately 10 minutes. She was immediately brought to hospital by relatives and there was no prehospital intervention. On arrival, in the emergency room, she was highly irritable and had impaired consciousness with a Glasgow Coma Scale (GCS) rating of 10. Bilateral pupils were normal in size and reaction and there was no neurological deficit. Heart rate was 45–50 beats per minute with a mean arterial pressure (MAP) of 120–130 mm Hg, respiratory rate was 18 per minute, and temperature was normal. The chest was bilaterally clear on auscultation. The optic nerve sheath diameter (ONSD) on orbital ultrasonography (USG) was 6.2 mm. Her body mass index was 24.2. She had no history of taking oral contraceptives, hematologic disorders, autoimmune diseases, or intracranial and extracranial tumors. An intravenous (IV) injection of 1 g levetiracetam and 20% mannitol was immediately started. IV labetalol infusion was started at the rate of 2 mg/min to maintain MAP < 90 mm Hg. The patient was admitted to the Medicine Department and shifted to the intensive care unit (ICU) for further management. Laboratory investigations including complete blood counts, coagulation profile, liver function, and kidney function tests were normal. On radiological imaging, noncontrast computed tomography (CT) was normal, but magnetic resonance venography (MRV) of the brain showed multiple acute infarcts in the bilateral cerebral hemisphere and dural venous thrombosis in the left transverse and sigmoid sinus suggestive of CVST (Figure 1).

The patient was started on subcutaneous injection of low-molecular-weight heparin (LMWH) (Enoxaprin) 1 mg/kg/body weight twice a day, IV levetiracetam 500 mg twice a day, and IV 20% mannitol thrice a day. Labetalol infusion was continued. Over the next three days, the patient showed gradual symptomatic improvement. She was no longer irritable, GCS improved to 15, and vomiting and headache also subsided completely. ONSD on orbital USG on her third day in the ICU was 3.2 mm. Labetalol was tapered off and Mannitol was stopped. She was shifted to tablet warfarin with a target internationalized normalized ratio (INR) of 2.5 (range 2–3), which was to be continued for six months and tablet levetiracetam 500 mg twice a day for a year. The patient was discharged after seven days from the onset of symptoms and asked to come for follow-up after two weeks and thereafter on a regular basis for one year.

## Discussion

CVST is a rare condition with a fatal outcome if not diagnosed and treated early. It accounts for 0.5–1% of all cases of stroke.^6^ The reported incidence of developing CVST during pregnancy and puerperium is 0.018–0.2%.^3^

CVST is associated with known risk factors such as thrombophilia, ear infections, cancer, head trauma, pregnancy, puerperium, dehydration, and oral contraceptive pills.^3-9,10-12^ There is a cause-and-effect relationship between the prothrombotic state and CVST. Pregnancy induces several prothrombotic changes in the coagulation system that persist for 6-eight weeks after birth.^7^ Volume depletion and trauma of delivery worsen the hypercoagulability. Cesarean section and infection during the puerperium are the additional risk factors for CVST.^8^ Our patient was a young post-partum woman who had undergone a cesarean section for delivery.

The symptoms of CVST depend on the sinuses and veins involved, raised intracranial pressure, and the extent of brain parenchymal injury. The superior sagittal sinus is the most frequently affected area (62%) followed by the transverse sinus.^1^ Headache is the most common presentation, typically described as diffuse and progressive in severity over days to weeks, not relieved by analgesics. It may be associated with diplopia and vomiting.^2^ Other symptoms may include sensorial loss, vertigo, dizziness, seizures, hemiparesis, and loss of consciousness.^9^ Our patient presented with severe headaches, vomiting, and convulsions. A detailed record of antenatal checkups and operative notes showed normal blood pressure throughout the pregnancy and during delivery, which ruled out postpartum eclampsia. Absence of headache for 10 days after dural puncture also ruled out PDPH.^5^

Patients with suspected CVST require neuroimaging to confirm the diagnosis. Noncontrast CT scan of the head is usually the first test, which may be normal in 30% of patients, or it may show specific signs, including venous sinus or deep vein hyperdensity.^13^ MRV or CT venography is required to confirm the diagnosis. Our patient had a normal CT of the head but showed left transverse and sigmoid sinus thrombosis on MRV, which was done due to a high level of clinical suspicion.

Treatment should be started, with anticoagulant therapy, control of seizures, and management of intracranial hypertension. Treatment of any reversible underlying cause such as dehydration, sepsis, should be started and any prothrombotic medications should be stopped.

LMWH is the preferred anticoagulant for CVST.^1^ Initial anticoagulation with a therapeutic dose of LMWH is followed by longer-term anticoagulation with warfarin (target INR is 2.5, range 2.0–3.0) to prevent further venous thrombotic events. It is recommended that patients with one episode of CVST and transient risk factors should receive anticoagulation for three to six months; patients with one episode of CVST of unknown cause should continue anticoagulation for 6–12 months; and patients with two or more episodes of CVST (or one episode and a severe prothrombotic condition with a high ongoing thrombotic risk) are recommended to have lifelong anticoagulation.^10^ Endovascular treatment by locally administrating fibrinolytic agents or mechanically removing it should be considered only in selective cases.^11^

Medical therapy for elevated intracranial pressure includes raising the head of the bed, osmotic therapy by mannitol, and hyperventilation (target PCO_2_ 30–35 mm Hg).^12^ Therapeutic lumbar puncture and decompressive craniectomy have also been proposed to reduce intracranial pressure in patients with midline shift.^14^

Seizures, if present, are to be treated with an anti-epileptic drug. For seizures associated with edema, infarction, or hemorrhage, anti-epileptic should be continued for at least one year.^15^

CVST generally has a favorable outcome, with a complete functional recovery in about 75% of patients. In women with a previous CVST, the absolute risk of pregnancy-related venous thrombosis is low but the relative risk of recurrence of CVST is 80-fold higher than the risk in the general population.^16^ Women with a history of CVST should be informed of the risks associated with future pregnancies.

## Conclusion

The case highlights that CVST should be included in the differential diagnosis if a woman presents with continuous headaches or seizures during pregnancy and the post-partum period. High clinical suspicion and the availability of imaging modalities such as MRV have contributed to the early diagnosis and a favorable outcome in this case. LMWH is the main stay of treatment in this condition.

## Author Contributions

SJ: Patient management, Conceptualization of case study, Collection of study material, and Manuscript Drafting. MB: Patient management and Manuscript drafting. VT: Patient management and Manuscript – revision & editing. All authors approved the final version of the manuscript.

## Competing Interests

The authors declare no competing interests.

## Informed Consent

Written informed consent was obtained from the patient for participating in our study.

## Patient Perspective

The patient was satisfied and pleased with the care she received throughout the therapy.
